# Sequential treatment of afatinib and osimertinib or other regimens in patients with advanced non‐small‐cell lung cancer harboring EGFR mutations: Results from a real‐world study in South Korea

**DOI:** 10.1002/cam4.4127

**Published:** 2021-07-13

**Authors:** Taeyun Kim, Tae Won Jang, Chang Min Choi, Mi‐Hyun Kim, Sung Yong Lee, Cheol‐Kyu Park, Yoon Soo Chang, Kye Young Lee, Seung Joon Kim, Sei Hoon Yang, Jeong Seon Ryu, Jeong Eun Lee, Shin Yup Lee, Chan Kwon Park, Sang Hoon Lee, Seung Hun Jang, Seong Hoon Yoon

**Affiliations:** ^1^ Department of Internal Medicine The Armed Forces Goyang Hospital Goyang‐si Korea; ^2^ Department of Internal Medicine Kosin University College of Medicine Kosin University Gospel Hospital Busan Korea; ^3^ Department of Internal Medicine Ulsan University Asan Medical Center Seoul Korea; ^4^ Department of Internal Medicine Pusan National University School of Medicine and Biomedical Research Institute Pusan National University Hospital Pusan Korea; ^5^ Division of Pulmonology, Allergy, and Critical Care Medicine Department of Internal Medicine Korea University Guro Hospital Seoul Korea; ^6^ Department of Internal Medicine Chonnam National University Hwasun Hospital Hwasun Korea; ^7^ Department of Internal Medicine Yonsei University Gangnam Severance Hospital Seoul Korea; ^8^ Department of Internal Medicine Konkuk University Medical Center Seoul Korea; ^9^ Department of Internal Medicine Catholic University Seoul St. Mary's Hospital Seoul Korea; ^10^ Department of Internal Medicine Wonkwang University Hospital Iksan Korea; ^11^ Department of Internal Medicine Inha University Hospital Incheon Korea; ^12^ Department of Internal Medicine Chungnam National University Hospital Daejeon Korea; ^13^ Department of Internal Medicine Kyungpook National University Chilgok Hospital Daegu Korea; ^14^ Department of Internal Medicine Catholic University Yeouido St. Mary's Hospital Seoul Korea; ^15^ Department of Internal Medicine Yonsei University Severance Hospital Seoul Korea; ^16^ Department of Internal Medicine Hallym University Sacred Heart Hospital Anyang Korea; ^17^ Department of Internal Medicine Pusan National University Yangsan Hospital Yangsan Korea

**Keywords:** afatinib, EGFR, NSCLC, osimertinib, real‐world data

## Abstract

**Objectives:**

The optimal sequence for the administration of epidermal growth factor receptor (EGFR) tyrosine kinase inhibitors (TKIs) for treating non‐small cell lung cancer (NSCLC) is still unclear. This study aimed to evaluate the efficacy of sequential afatinib and osimertinib treatment in patients with NSCLC harboring EGFR mutations.

**Materials and methods:**

Electronic records of patients with EGFR‐mutated NSCLC, who were administered afatinib and osimertinib (group A) or other chemotherapy (group B) between October 2014 and 2019, across 16 hospitals in South Korea were reviewed. The primary outcome, time on treatment (TOT), secondary outcome, and overall survival (OS) were estimated using the Kaplan–Meier method and log‐rank test. Multivariate analyses were performed using the Cox proportional hazards model.

**Results:**

Of the 737 patients who received frontline afatinib treatment, 324 with complete records were selected (group A: 126, group B: 198). All patients in group A were T790M positive after afatinib, while patients in group B were all negative or unknown. The median TOT was 35.4 months (95% confidence interval [CI]: 27.7−45.6) in group A and 20.8 months (95% CI: 19.4−24.0) in group B. The median TOT with afatinib was 13.0 months (95% CI: 12.0−13.9) overall and 15.7 months (95% CI: 13.9−17.3) in group A. The 2‐ and 3‐year survival rates were 86.0 and 69.3% in group A and 75.9 and 55.3% in group B, respectively.

**Conclusion:**

Sequential afatinib and osimertinib treatment resulted in better survival rates than treatment with afatinib followed by other chemotherapies.

## Graphical abstract

Our study evaluated the real‐world efficacy of sequential treatment of afatinib and osimertinib, and compared it with other chemotherapies as a second‐line treatment. Promising real‐world data on first‐line afatinib and second‐line osimertinib may maximize sustained clinical benefit with avoiding chemotherapy and may warrant consideration, though tailored personalized approaches are necessary.

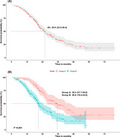

## 1 INTRODUCTION

Lung cancer is the leading cause of cancer‐related deaths in Korea, accounting for approximately 20% of all cancer‐related deaths; in 2018, 19,317 people died from the disease.^1^ Globally, 18.4% of all cancer‐related deaths were attributable to lung cancer and approximately 2 million people were newly diagnosed in 2018.^2^

Afatinib is a second‐generation epidermal growth factor receptor (EGFR) tyrosine kinase inhibitor (TKI) that is suggested as the primary treatment option for progressive, EGFR‐mutated NSCLC.^3^ A recent head‐to‐head trial demonstrated that afatinib was superior to gefitinib, a first‐generation TKI, showing better progression‐free survival (PFS) and time‐to‐treatment failure (TTF).^4^ Despite having many advantages over standard platinum‐based chemotherapy and first‐generation EGFR TKIs, mutations that confer resistance to afatinib are important clinical problems. The T790M resistance mutation, which is found in exon 20 of the *EGFR* gene, has been identified in approximately 50% of patients receiving afatinib as first‐line therapy.^5^ The AURA study showed a high objective response rate (ORR) and encouraging PFS with the third‐generation EGFR TKI, osimertinib, in patients with T790M‐mutated NSCLC previously treated with another TKI.^6^ Based on studies to date, the expected median period for sequential TKI treatment is approximately 24 months, 13 to 14 months with afatinib, and 10 to 13 months with osimertinib.^4^, ^6^, ^7^

Real‐world data (RWD) are generally used to monitor post‐market safety and adverse events. RWD additionally allow clinical decision‐making on patient groups that have been excluded by the generally strict inclusion/exclusion criteria of randomized clinical trials (RCTs). Such patients include elderly patients, those with poor performance status, those harboring uncommon *EGFR* mutations (i.e., neither the exon 19 deletion [Del19] nor L858R), and those with brain metastases. Thus, RWD may better reflect actual clinical conditions. The GioTag study, a retrospective, observational, and global multicenter study of NSCLC patients who received sequential treatment with afatinib and osimertinib, revealed a median TKI‐therapy period of 27.6 months and found that patients with the exon 19 mutation were appropriate candidates for these TKI therapies.^8^ In a subgroup analysis of 50 Asian patients, the median time on TKI therapy was ~46.7 months. Other RWD from South Korea indicated that the median PFS was 19.1 months for afatinib, 13.7 months for gefitinib, and 14.0 months for erlotinib, respectively.^9^

Several studies have been conducted in Asian populations on the cumulative advantage of frontline treatment with afatinib in a real‐world setting in NSCLC patients harboring *EGFR* mutations.^9^, ^10^, ^11^, ^12^, ^13^However, there is still a paucity of RWD regarding the clinical characteristics and outcomes of patients who were treated sequentially with afatinib and osimertinib. Moreover, considering the controversy on whether osimertinib should be used as first‐line TKI therapy or second‐line TKI therapy after the failure of first‐or second‐generation TKIs, the acquisition of data comparing first‐line afatinib followed by second‐line osimertinib with other second‐line treatments is warranted. In this context, the present study evaluated the clinical characteristics and treatment outcomes in patients with EGFR‐mutated NSCLC who received first‐line treatment with afatinib and second‐line therapy with either osimertinib or other regimens by analyzing RWD in South Korea.

## 2 MATERIALS AND METHODS

### 2.1 Patients, design, and data collection

Real‐world Experience of sequential treatment of afatinib and osimertinib (RESET) is a retrospective multicenter observational study in South Korea across 16 medical centers. Electronic medical records from October 2014 to 2019 of patients who met the following inclusion criteria were reviewed: (ⅰ) age ≥19 years with EGFR‐mutated TKI‐naïve advanced‐stage NSCLC that was newly diagnosed pathologically and (ii) treated first‐line with afatinib and second‐line with either osimertinib or other treatments. The NSCLC advanced stages were defined as stages 3B, 3C, 4A, and 4B, which are not eligible for standard operative procedures, based on the 8th edition of the American Joint Committee on Cancer (AJCC) staging system. Patients who were not treated with afatinib as first‐line therapy or osimertinib as second‐line therapy were excluded. Patients who were initially treated with chemoradiotherapy were also excluded.

A total of 737 patients with EGFR‐mutated advanced‐stage NSCLC were enrolled in the study. Of these, we excluded 413 patients: 164 patients continued afatinib therapy, 110 experienced progressive disease with no available data on second‐line treatment, 10 refused treatment, 15 discontinued treatment due to afatinib toxicity, 68 were transferred or lost to follow‐up, and 36 without information on T790M mutation. A final set of 324 eligible patients were selected. Patients who received osimertinib as a second‐line treatment and presented T790M mutation after afatinib were categorized into group A (*n* = 126). Patients who received other regimens as a second‐line treatment and did not present or prove T790M mutation after afatinib were categorized into group B (*n* = 198). The study flow chart is depicted in Figure [Link], [Link], [Link], [Link].

Baseline demographic characteristics (age, sex, and Eastern Cooperative Oncology Group performance status [ECOG PS]) were collected. Smoking status was categorized into never, former, and current smokers according to the classification of the National Health Interview Survey. The date of diagnosis, initiation of first‐line afatinib, information on EGFR mutation (i.e., presence/absence and profile), number of metastatic organs, existence of specific organ metastasis, and dose modification of afatinib were recorded. For EGFR mutation analysis, the peptic nucleic acid‐mediated real‐time polymerase chain reaction (PCR) clamping method (Panagene, Daejeon, Korea) or the Roche Cobas EGFR mutation test (Roche Molecular Systems, Pleasanton, CA, USA) was used. Follow‐up data regarding the date and regimen of second‐line treatment and new lesions or aggravation of brain metastasis were also collected.

### 2.2 Ethical approval

The study and protocol were approved by the Institutional Review Board of the Kosin University Gospel Hospital (KUGH no. 2019‐07‐038). The study was conducted following the Declaration of Helsinki. All procedures were performed in accordance with the relevant guidelines and regulations.

### 2.3 Outcomes and measurements

The primary outcome was time on treatment (TOT). TOT‐1 was defined as the time from the first dose of afatinib to tumor progression, TOT‐2 was defined as the time from the first dose of second‐line therapy to tumor progression or death during the treatment, and overall TOT was defined as the length between the first dose of afatinib and tumor progression or death during the second‐line treatment.

The secondary outcomes were as follows: (ⅰ) ORR‐1, defined as the ratio of total patients who received afatinib to patients experiencing complete remission (CR) or partial remission (PR) after the first evaluation of tumor response, which was defined based on Response Evaluation Criteria in Solid Tumors (RECIST) version 1.1,^14^ (ii) ORR‐2 for second‐line treatment, (iii) disease control rate‐1 (DCR‐1), defined as the ratio of total patients receiving afatinib to patients with CR, PR, and stable disease (SD) after the first evaluation of tumor response defined based on RECIST version 1.1, (iv) DCR‐2 for osimertinib, and (ⅴ) overall survival (OS), defined as the length of time from the start of afatinib to death from any cause. Patients still on treatment were censored at the time of data collection.

### 2.4 Statistical analysis

The baseline patient characteristics were descriptive. Chi‐squared and Fisher’s exact tests were used to compare the differences between categorical variables. TOT and OS were estimated with the Kaplan–Meier method and differences in time distributions were compared using the log‐rank test; the estimated median time (months) and 95% confidence interval (CI) are presented. The Cox proportional hazards (PH) model was used to investigate the effect of independent variables on survival outcomes. Variables with *p* < 0.10 in the univariate Cox PH model were included in the multivariate Cox PH model. All statistical analyses were performed using IBM SPSS Statistics for Windows, version 25.0 (IBM Corp., Armonk, New York), and R software version 4.0.3 for Windows (R Development Core Team).

## 3 RESULTS

### 3.1 Patient characteristics

The baseline characteristics of the study participants are summarized in Table 1. The tumor stage was more advanced in group A patients than in group B patients. The latter group experienced a higher percentage of newly appeared or aggravated brain metastases. Other variables were not statistically different between groups A and B. At the start of second‐line treatments, the presence and type of brain metastasis were well balanced.

**TABLE 1:** Characteristics of study participants

	First‐line treatment	Second‐line treatment		
	Afatinib (*n* = 324)	Group A (*n* = 126)	Group B (*n* = 198)	*p*
Men	177 (54.6%)	70 (55.6%)	107 (54.0%)	0.789
Age				0.290
<65	181 (55.9%)	75 (59.5%)	106 (53.5%)	
≥65	143 (44.1%)	51 (40.5%)	92 (46.5%)	
Stage^§^				0.003
3 and 4A	189/323 (58.5%)	61 (48.4%)	128/197 (65.0%)	
4B	134/323 (41.5%)	65 (51.6%)	69/197 (35.0%)	
Smoking				0.922
Never	198/321 (61.7%)	76 (60.3%)	122/195 (62.6%)	
Former	91/321 (28.3%)	37 (29.4%)	54/195 (27.7%)	
Current	32/321 (10.0%)	13 (10.3%)	19/195 (9.7%)	
ECOG PS				
0 and 1	274/297 (92.3%)	101/106 (95.3%)	173/191 (90.6%)	0.178
≥2	23/297 (7.7%)	4/106 (4.7%)	18/191 (9.4%)	
Tissue type				
Adenocarcinoma	317 (97.8%)	123 (97.6%)	194 (98.0%)	0.828
Others^¶^	7 (2.2%)	3 (2.4%)	4 (2.0%)	
Presence of EGFR mutation	324 (100%)	126 (100%)	198 (100%)	‐
EGFR mutation				0.138
Del19	178 (54.9%)	77 (61.1%)	101 (51.0%)	
L858R	100 (30.9%)	36 (28.6%)	64 (32.3%)	
Others^†^	46 (14.2%)	13 (10.3%)	33 (16.7%)	
# of metastatic organs				0.058
0–1	153 (47.2%)	51 (40.5%)	102 (51.5%)	
2–3	140 (43.2%)	58 (46.0%)	82 (41.4%)	
4 or more	31 (9.6%)	17 (13.5%)	14 (7.1%)	
Presence of adrenal gland meta.	25 (7.7%)	11 (8.7%)	14 (7.1%)	0.585
Presence of liver meta.	45 (13.9%)	23 (18.3%)	22 (11.1%)	0.070
Presence of bone meta.	139 (42.9%)	60 (47.6%)	79 (39.9%)	0.171
Presence of brain meta.	142 (43.8%)	54 (42.9%)	88 (44.4%)	0.779
Type of brain meta.				0.961
Single parenchymal	21/140 (15.0%)	8/54 (14.8%)	13/86 (15.1%)	
Multiple +/− seeding	119/140 (85.0%)	46/54 (85.2%)	73/86 (84.9%)	
New lesion or aggravation of brain meta.				0.035
Yes	85/323 (26.3%)	25/126 (19.8%)	60/197 (30.5%)	
No	238/323 (73.7%)	101/126 (80.2%)	137/197 (69.5%)	
Dose adj. for afatinib				0.115
Yes	206/323 (63.8%)	87 (69.0%)	119/197 (60.4%)	
No	117/323 (36.2%)	39 (31.0%)	78/197 (39.6%)	

Data are presented as numbers (percentages), unless otherwise stated.

Patients in group A received sequential treatment with afatinib and osimertinib, while patients in group B received other therapies following first‐line afatinib treatment.

Abbreviations: adj., adjustment; Del19, deletion 19; ECOG PS, Eastern Cooperative Oncology Group performance status; EGFR, epidermal growth factor receptor; meta., metastasis.

§ Tumor stage was classified based on 8th edition of the American Joint Committee on Cancer staging system.

¶ Other tissue types included squamous cell carcinoma in two patients, adenosquamous cell carcinoma in 0 patients, and non‐small cell lung cancer in three patients.

† Patients not presenting with EGFR Del19 and L858R mutations, including de novo T790M mutation, are classified as the “Others” group.

### 3.2 Median TOT during first‐ and second‐line treatments

**FIGURE 1:**
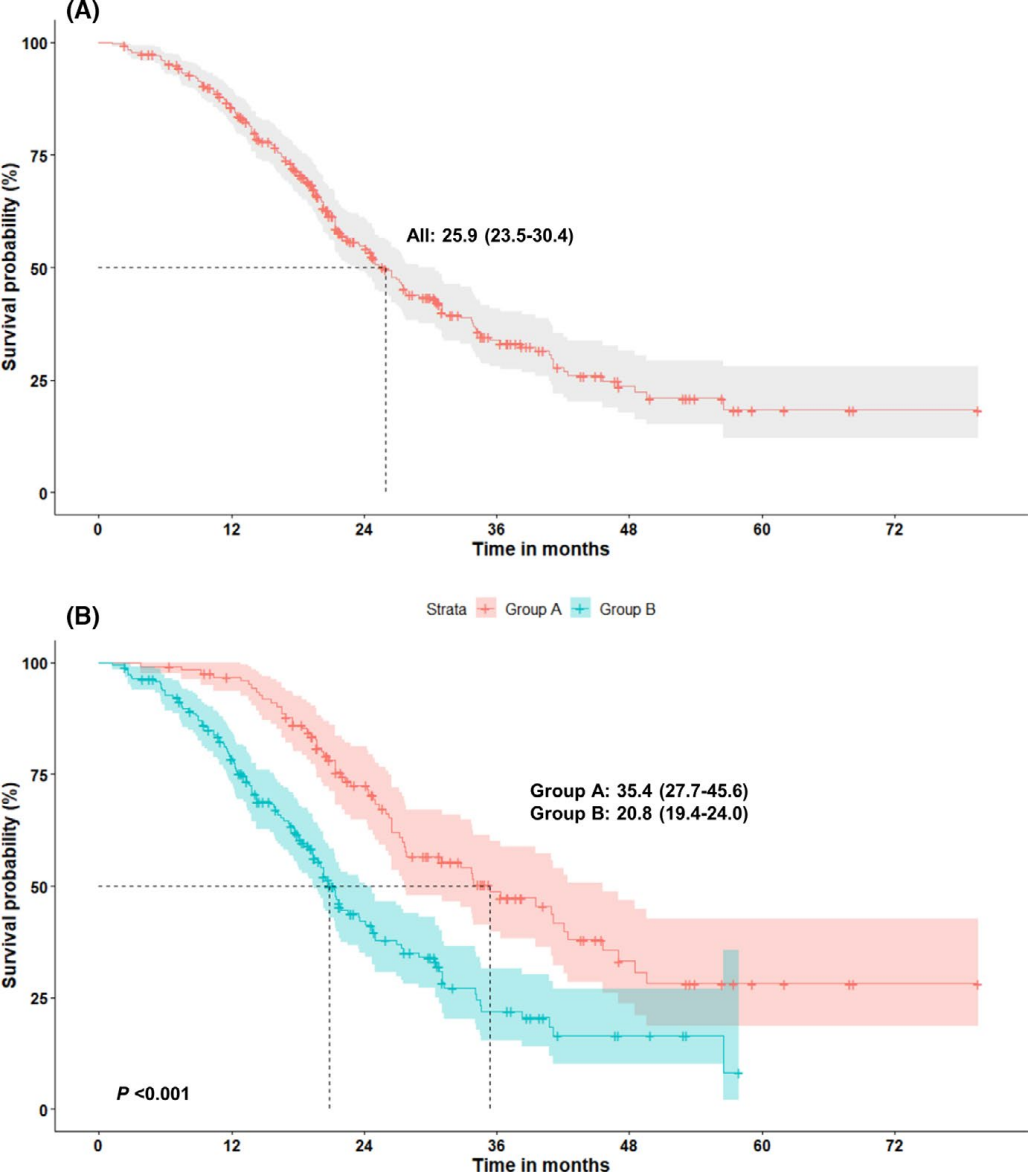
(A) Overall time‐on‐treatment (TOT) for first‐ and second‐line treatments in all patients (*n* = 324) with advanced NSCLC harboring EGFR mutations. (B) Overall TOT, using osimertinib (group A, *n* = 126) and other medications (group B, *n* = 198) as second‐line treatment

The results of median TOT during the first‐ and second‐line treatments are summarized in Table 2. The median TOT in all patients was estimated to be 25.9 (95% CI: 23.5–30.4) months. The overall TOT in group A was 35.4 (95% CI: 27.7–45.6) months, which was significantly longer than that in group B (20.8 [95% CI: 19.4–24.0] months, *p* < 0.001, Figure 1).

**TABLE 2:** Overall median time‐on‐treatment in patients who received first‐ and second‐line treatments

	Total (*n* = 324)	*p*	Group A (*n* = 126)	*p*	Group B (*n* = 198)	*p*
Overall	25.9 (23.5–30.4)		35.4 (27.7–45.6)		20.8 (19.4–24.0)	
Age		0.627		0.662		0.546
<65	26.4 (22.7–31.0)		33.8 (26.4–NA)		21.7 (19.9–30.5)	
≥65	24.2 (20.8–33.7)		41.1 (31.0–NA)		20.3 (18.0–23.5)	
Sex		0.267		0.671		0.047
Men	24.4 (21.3–30.4)		36.4 (26.2–NA)		19.4 (16.7–21.7)	
Women	27.6 (25.0–33.9)		35.4 (27.6–48.5)		24.0 (20.5–31.0)	
ECOG PS		0.023		0.667		0.092
0 or 1	26.5 (24.2–31.0)		33.9 (27.7–47.0)		21.5 (19.9–25.0)	
≥2	19.8 (14.2–NA)		20.1 (14.2–NA)		16.9 (11.5–NA)	
Stage^§^		0.251		0.029		0.294
3 and 4A	27.4 (24.0–34.2)		41.1 (35.4–NA)		21.5 (19.4–27.4)	
4B	22.0 (21.3–27.1)		26.5 (24.4–NA)		19.9 (17.6–21.9)	
Smoking		0.867		0.584		0.175
Never	26.9 (24.7–31.0)		33.9 (27.4–48.5)		22.5 (20.3–30.4)	
Former	24.7 (21.4–32.8)		33.8 (25.3–NA)		20.3 (17.6–27.4)	
Current	22.2 (14.3–NA)		47.0 (22.7–NA)		12.3 (8.9–NA)	
Tissue type		0.004		0.131		0.001
Adenocarcinoma	26.2 (23.5–30.8)		35.4 (27.7–45.6)		21.4 (19.8–24.7)	
Others^¶^	14.7 (8.9–NA)		27.1 (14.7–NA)		10.5 (5.5–NA)	
EGFR mutation		0.813		0.659		0.136
Del19	26.9 (23.5–31.0)		40.9 (27.8–49.5)		21.4 (20.0–26.9)	
L858R	26.2 (21.4–34.0)		26.5 (25.0–NA)		24.0 (19.4–NA)	
Others^†^	23.5 (17.1–47.0)		39.5 (33.9–NA)		17.2 (13.4–24.7)	
# of metastatic organs		0.005		0.029		0.004
0–1	30.8 (25.0–40.9)		45.6 (39.5–NA)		24.7 (21.5–34.0)	
2–3	21.7 (20.1–27.5)		31.0 (26.4–42.4)		19.4 (16.4–21.4)	
4 or more	20.3 (16.2–48.5)		24.4 (19.6–NA)		16.2 (13.0–NA)	
Adrenal gland meta.		0.963		0.942		0.677
Yes	25.3 (23.5–29.0)		33.9 (27.4–45.6)		16.2 (10.7–NA)	
No	32.8 (18.8–NA)		41.1 (32.8–NA)		21.4 (19.8–24.7)	
Liver meta.		<0.001		<0.001		<0.001
Yes	19.1 (15.5–24.4)		21.4 (19.1–32.8)		13.8 (7.4‐NA)	
No	27.5 (24.8–33.9)		42.1 (33.9–NA)		21.4 (20.0–25.0)	
Bone meta.		0.012		0.035		0.006
Yes	21.4 (20.1–25.9)		27.7 (24.4–41.1)		19.4 (17.2–20.8)	
No	29.0 (25.3–35.4)		42.1 (35.4–NA)		23.5 (20.3–31.0)	
Brain meta.		0.482		0.543		0.732
Yes	24.4 (21.4–30.4)		33.7 (25.3‐NA)		21.7 (19.4–30.5)	
No	27.1 (23.5–33.8)		36.4 (27.4–49.5)		20.3 (18.0–24.7)	
Type of brain meta.		0.534		0.812		0.276
Single parenchymal	25.3 (20.3–NA)		42.4 (13.5–NA)		21.4 (20.3–NA)	
Multiple +/− seeding	24.4 (21.4–29.0)		33.7 (24.8–NA)		19.4 (17.1–24.9)	
New lesion or aggravation of brain meta.		0.005		0.148		0.127
Yes	21.4 (18.0–25.9)		27.1 (20.1–49.5)		19.9 (14.3–23.5)	
No	27.6 (25.0–33.8)		39.5 (31.0–48.5)		21.7 (19.8–29.0)	
Dose adj. for afatinib		0.047		0.503		0.072
Yes	27.4 (23.5–33.9)		35.4 (27.6–49.5)		21.7 (20.3–30.5)	
No	25.0 (19.7–29.0)		33.8 (26.5–NA)		18.9 (15.8–24.9)	

Data are presented as months (95% confidence intervals), unless otherwise stated.

Patients in group A received sequential treatment with afatinib and osimertinib, while patients in group B received other therapies following first‐line afatinib treatment.

Abbreviations: adj., adjustment; Del19, deletion 19; ECOG PS, Eastern Cooperative Oncology Group performance status; EGFR, epidermal growth factor receptor; meta., metastasis; NA, not‐available.

§ Tumor stage was classified based on 8th edition of the American Joint Committee on Cancer staging system.

¶ Other tissue types included squamous cell carcinoma in two patients, adenosquamous cell carcinoma in two patients, and non‐small cell lung cancer in three patients.

† Patients not presenting with EGFR Del19 and L858R mutations, including de novo T790M mutation, are classified as the “Others” group.

### 3.3 Respective median TOT (TOT‐1 and TOT‐2) according to first‐ and second‐line treatments

**FIGURE 2:**
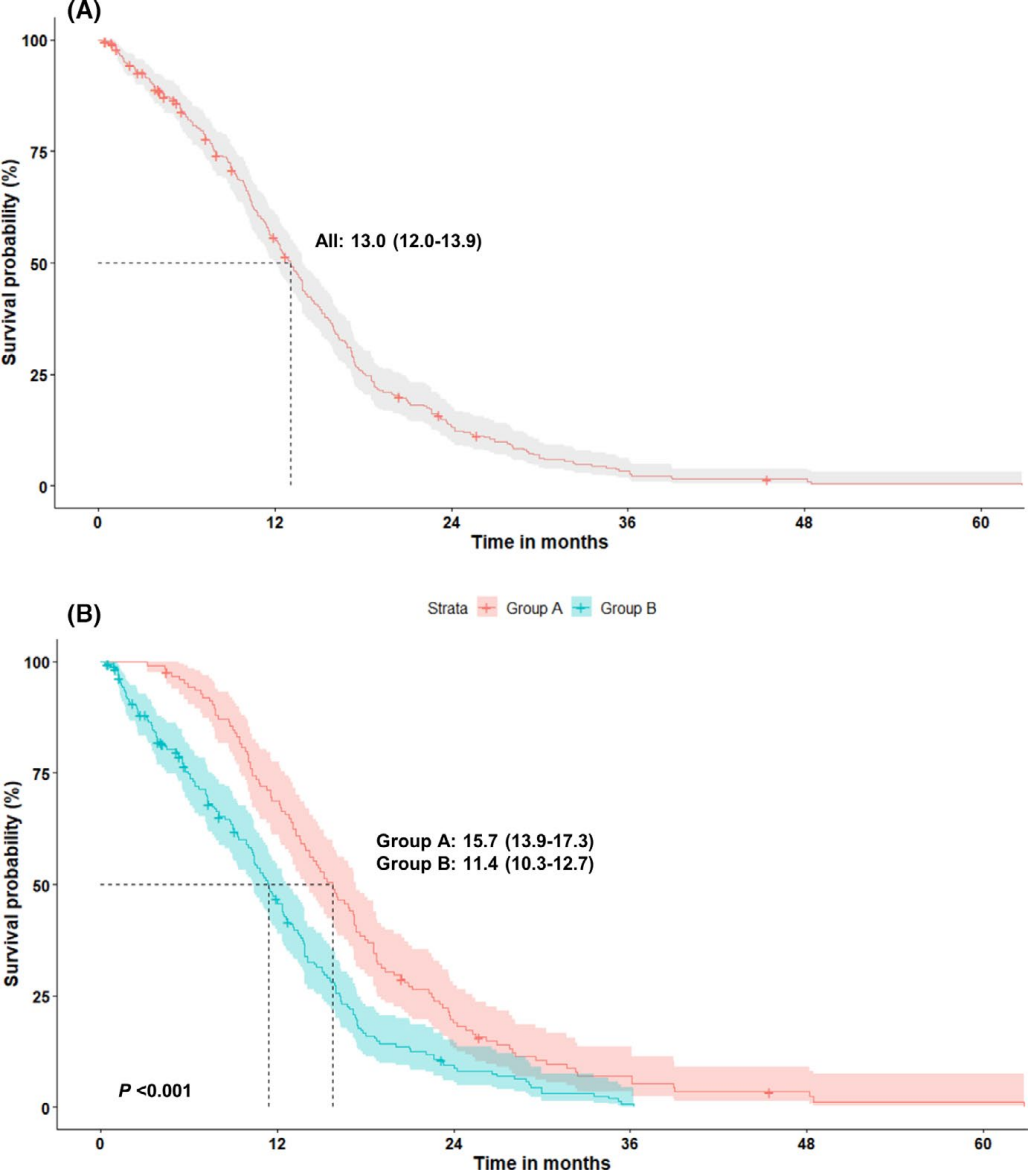
(A) Time‐on‐treatment (TOT) for first‐line treatment with afatinib in all patients (*n* = 324) with advanced NSCLC harboring EGFR mutations. (B) TOT with afatinib, when osimertinib (group A, *n* = 126) and other medications (group B, *n* = 198) are used as second‐line treatment

The individual median TOTs for the first‐ and second‐line treatments are depicted in Table 3. The estimated median TOT‐1 was 13.0 (95% CI: 12.0–13.9) months in all patients, 15.7 (95% CI: 13.9–17.3) months in group A patients, and 11.4 (95% CI: 10.3–12.7) months in group B patients. The difference in TOT‐1 between groups A and B was statistically significant (*p* < 0.001, Figure 2). The median TOT‐2 in groups A and B patients was 11.9 (95% CI: 10.2–26.5) and 5.1 (95% CI: 4.1–6.9) months, respectively, and this difference was significant (*p* < 0.001).

**TABLE 3:** Respective median time‐on‐treatment in patients who received first‐ and second‐line treatments

	First‐line treatment						Second‐line treatment			
	Total (*n* = 324)	*p*	Group A (*n* = 126)	*p*	Group B (*n* = 198)	*p*	Group A (*n* = 126)	*p*	Group B (*n* = 198)	*p*
Overall	13.0 (12.0–13.9)		15.7 (13.9–17.3)		11.4 (10.3–12.7)		11.9 (10.2–26.5)		5.1 (4.1–6.9)	
Age		0.324		0.429		0.039		0.888		0.546
<65	13.8 (12.4–15.5)		15.9 (13.9–17.9)		11.4 (10.2–14.0)		11.2 (8.5–NA)		4.8 (3.0–8.4)	
≥65	12.0 (10.8–13.8)		15.1 (11.6–20.9)		11.4 (8.9–12.9)		13.4 (10.3–NA)		5.5 (4.2–9.4)	
Sex		0.343		0.592		0.022		0.901		0.270
Men	12.5 (11.4–13.9)		15.8 (13.2–17.6)		10.4 (9.0–12.5)		12.9 (8.4–NA)		4.8 (3.6–6.3)	
Women	13.6 (12.0–15.4)		15.2 (13.6–19.4)		12.4 (10.6–15.2)		11.2 (10.2–NA)		6.7 (3.7–10.2)	
ECOG PS		0.014		0.044		0.229		0.610		0.287
0 or 1	13.2 (12.3–14.4)		15.7 (13.8–17.3)		11.6 (10.4–13.5)		11.2 (9.0–NA)		5.1 (4.1–7.0)	
≥2	9.9 (7.7–13.6)		8.0 (6.4–NA)		10.2 (7.7–17.6)		13.0 (5.9–NA)		4.9 (2.3–NA)	
Stage^§^		<0.001		0.001		0.027		0.492		0.882
3 and 4A	14.2 (12.4–15.9)		17.9 (16.8–23.0)		12.0 (10.4–14.1)		13.4 (8.5–NA)		5.9 (4.1–8.4)	
4B	12.0 (10.5–13.2)		13.6 (12.3–15.8)		10.3 (7.9–12.4)		11.2 (9.0–NA)		4.9 (3.5–10.9)	
Smoking		0.583		0.453		0.085		0.657		0.756
Never	13.5 (12.2–14.8)		15.2 (13.5–17.6)		12.0 (10.6–13.8)		11.2 (10.2–NA)		5.9 (3.6–9.4)	
Former	12.8 (11.4–16.1)		16.1 (11.5–18.8)		11.6 (8.4–15.9)		11.9 (8.1–NA)		4.6 (3.9–10.3)	
Current	12.3 (7.9–15.4)		15.4 (13.2–NA)		5.5 (3.6–13.8)		NA (8.44–NA)		6.3 (2.6–NA)	
Tissue type		0.032		0.159		0.066		0.305		0.091
Adenocarcinoma	13.1 (12.2–14.1)		15.8 (14.2–17.3)		11.4 (10.4–12.7)		12.9 (10.3–26.5)		5.5 (4.2–7.0)	
Others^¶^	10.3 (3.1–NA)		10.7 (10.3–NA)		5.6 (2.4–NA)		9.4 (3.5–NA)		2.6 (2.0–NA)	
EGFR mutation		0.681		0.626		0.714		0.749		0.832
Del19	13.7 (12.8–15.2)		15.8 (13.9–17.3)		12.4 (10.6–13.9)		11.9 (9.4–NA)		4.9 (3.6–8.4)	
L858R	11.6 (10.0–15.0)		15.7 (11.5–20.4)		9.3 (6.7–13.9)		11.2 (8.3–NA)		5.5 (3.6–NA)	
Others^†^	11.6 (9.7–13.8)		13.2 (11.6–NA)		10.8 (5.6–12.6)		20.0 (7.1–NA)		6.2 (2.6–17.1)	
# of metastatic organs		0.015		0.038		0.081		0.128		0.003
0–1	14.6 (12.6–15.9)		17.2 (15.2–20.4)		12.3 (10.8–15.0)		26.5 (10.3–NA)		7.0 (4.9–13.6)	
2–3	12.6 (10.8–13.8)		14.4 (13.0–18.5)		10.4 (8.9–13.1)		13.0 (9.1–NA)		4.3 (2.9–6.3)	
4 or more	11.2 (8.0–13.9)		12.8 (8.7–22.3)		10.0 (7.1–17.1)		8.6 (5.9–NA)		2.3 (2.1–NA)	
Adrenal gland meta.		0.448		0.519		0.053		0.871		0.721
Yes	11.2 (7.8–18.8)		15.4 (13.8–17.2)		5.3 (3.4–NA)		11.0 (5.9–NA)		2.7 (2.1–NA)	
No	13.1 (12.2–14.1)		18.5 (13.6–NA)		11.6 (10.4–13.4)		11.9 (9.1–NA)		5.5 (4.2–7.0)	
Liver meta.		0.005		0.008		0.015		0.001		0.003
Yes	10.0 (7.8–13.6)		11.5 (9.1–18.8)		7.1 (3.8–14.1)		8.4 (5.9–11.9)		2.0 (1.4–NA)	
No	13.5 (12.4–14.7)		16.8 (14.8–18.1)		11.6 (10.5–13.4)		17.3 (11.2–NA)		6.2 (4.6–8.4)	
Bone meta.		0.059		0.227		0.040		0.099		0.049
Yes	12.4 (11.2–13.8)		14.0 (12.8–17.3)		11.0 (8.7–12.7)		11.0 (8.4–13.4)		4.4 (2.9–6.7)	
No	13.6 (12.3–15.4)		16.9 (14.8–18.5)		11.4 (10.4–13.8)		20.0 (9.4NA)		6.3 (4.2–10.4)	
Brain meta.		0.263		0.248		0.949		0.737		0.688
Yes	12.4 (11.2–13.6)		13.6 (12.3–18.5)		11.6 (10.2–13.4)		13.0 (8.4–NA)		5.5 (3.9–8.7)	
No	13.9 (11.8–15.5)		16.8 (15.5–18.5)		11.3 (9.3–13.8)		11.2 (9.0–NA)		4.9 (3.6–9.1)	
Type of brain meta.		0.337		0.983		0.115		0.974		0.548
Single parenchymal	16.2 (8.7–22.6)		18.5 (5.7–NA)		12.7 (8.7–NA)		17.3 (4.4–NA)		6.3 (2.8–NA)	
Multiple +/− seeding	12.4 (10.8–13.6)		13.4 (12.3–17.3)		11.2 (9.9–13.4)		11.9 (8.2–NA)		5.5 (3.9–8.7)	
New lesion or aggravation of brain meta.		0.758		0.347		0.545		0.078		0.003
Yes	11.6 (10.3–14.7)		15.9 (10.2–24.8)		11.0 (9.9–13.6)		10.3 (7.1–17.3)		3.4 (2.4–5.5)	
No	13.5 (12.4–14.6)		15.7 (14.2–17.3)		11.8 (10.0–13.7)		13.4 (10.6–NA)		6.4 (4.8–10.9)	
Dose adj. for afatinib		<0.001		0.175		<0.001		0.712		0.824
Yes	13.8 (12.6–15.4)		15.6 (13.1–18.7)		12.6 (11.3–15.0)		13.0 (10.3–NA)		5.9 (3.9–7.0)	
No	10.8 (10.2–13.2)		15.7 (13.8–17.6)		9.3 (7.3–11.6)		11.2 (8.5–NA)		4.9 (3.5–10.2)	

Data are presented as months (95% confidence intervals), unless otherwise stated.

Patients in group A received sequential treatment with afatinib and osimertinib, while patients in group B received other therapies following first‐line afatinib treatment.

Abbreviations: adj., adjustment; Del19, deletion 19; ECOG PS, Eastern Cooperative Oncology Group performance status; EGFR, epidermal growth factor receptor; meta., metastasis; NA, not‐available.

§ Tumor stage was classified based on 8^th^ edition of the American Joint Committee on Cancer staging system.

¶ Other tissue types included squamous cell carcinoma in two patients, adenosquamous cell carcinoma in two patients, and non‐small cell lung cancer in three patients.

† Patients not presenting with EGFR Del19 and L858R mutations, including de novo T790M mutation, are classified as the “Others” group.

### 3.4 Multivariate Cox PH analysis of factors affecting TOT‐1

**FIGURE 3:**
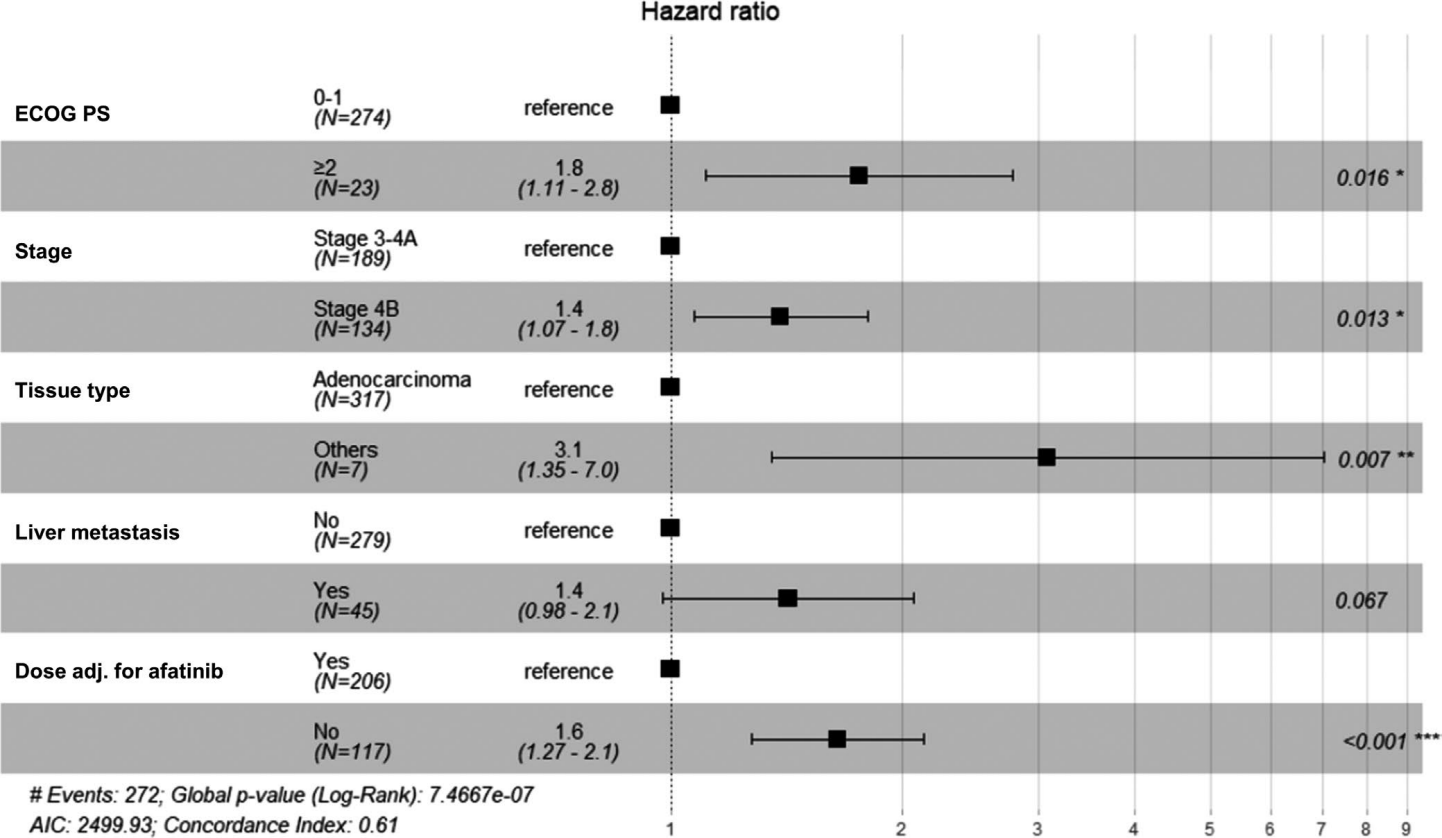
Multivariate Cox proportional hazards regression analysis of factors affecting time on afatinib treatment in all patients (*n* = 324) with advanced NSCLC harboring EGFR mutations

**FIGURE 4:**
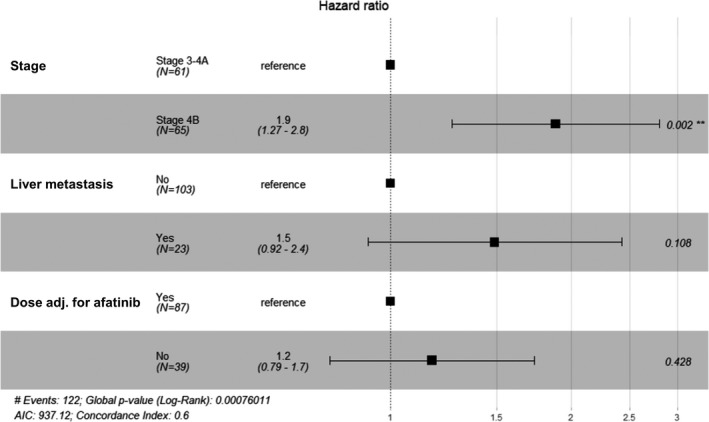
Multivariate Cox proportional hazards regression analysis of factors affecting the time on first‐line afatinib treatment in patients with advanced NSCLC, harboring EGFR mutations, and receiving osimertinib as a second‐line treatment (*n* = 126)

In all patients, the multivariate Cox PH model revealed that poor ECOG PS, advanced tumor stage, tissue type other than adenocarcinoma, presence of liver metastasis, and no afatinib dose adjustment were related to decreased TOT‐1 (Figure 3). In group A, the hazard ratio (HR) was higher in patients with advanced tumor stage, and the presence of liver metastasis was associated with a marginally significant decrease in TOT‐1 (Figure 4).

### 3.5 Multivariate Cox PH analysis of factors affecting TOT‐2 in group A patients

In group A patients who received second‐line osimertinib treatment, the presence of liver metastasis was associated with a decrease in TOT‐2 with an HR of 2.4 (95% CI: 1.35–4.40, *p* = 0.003, Figure [Link], [Link], [Link], [Link]).

### 3.6 ORR‐1 and DCR‐1

The results of ORR‐1 and DCR‐1 are shown in Tables S1 and S2, respectively. In the patients who received first‐line afatinib treatment, the ORR‐1 was 69.1% in all patients, 75.0% in group A, and 65.2% in group B. The DCR‐1 in all patients was 93.2%, with 97.6% in group A and 90.4% in group B.

### 3.7 ORR‐2 and DCR‐2

The results of ORR‐2 and DCR‐2 are shown in Tables S3 and S4, respectively. The ORR‐2 was 40.2% in group A and 20.5% in group B. DCR‐2 was higher than ORR‐2 in both groups. The DCR‐2 was 94.6% in group A and 75.2% in group B.

### 3.8 OS, 2‐year, and 3‐year survival rates

The estimated OS was 49.1 (95% CI: 39.4–58.8) months in all patients with 2‐year and 3‐year survival rates of 78.1 and 63.5%, respectively (Table [Link], [Link], [Link], [Link]). The median OS was not reached in group A patients and it was 38.5 (95% CI: 28.8–48.2) months in group B patients (Table [Link], [Link], [Link], [Link]). The OS was significantly longer in group A than in group B (*p* = 0.0016, Figure [Link], [Link], [Link], [Link]).

## 4 DISCUSSION

The present study compared the data of patients who received sequential treatment with afatinib and osimertinib to those of patients who received second‐line treatments other than osimertinib. We comprehensively investigated clinical outcomes (i.e., TOT, OS, DCR, and ORR) and robustly evaluated the factors affecting TOT in afatinib and osimertinib treatments. Considering the controversies regarding the use of first‐line osimertinib followed by other therapies or first‐line first‐/second‐generation TKIs followed by osimertinib as appropriate options for treating EGFR‐mutated NSCLC patients,^15^ the RWD from RESET might be of interest. RESET included a group of old‐age patients with poor performance status and brain metastases, characteristics likely to exclude these patients from RCTs due to strict inclusion criteria. Thus, the results from RESET could be applicable across real‐world clinical settings in the management of advanced‐stage EGFR‐mutated NSCLC patients.

RESET showed that clinical efficacy was better in group A patients than in group B patients. The estimated overall TOT in group A was 35.4 months, which was significantly longer than that in group B (20.8 months). Subgroup analyses also revealed that overall TOT was longer in group A than in group B for all subdivided clinical characteristics, such as smoking status or EGFR mutation type. If we separated first‐ and second‐line treatments, the median TOTs in group A patients were superior to those in group B patients. In addition to TOT, group A was numerically superior to group B with respect to OS, DCR, and ORR for all subdivided clinical characteristics. The AURA‐3 clinical trial demonstrated that second‐line osimertinib had significantly greater efficacy than pemetrexed plus platinum‐based therapy in advanced‐stage T790M‐mutated NSCLC during treatment with first‐line EGFR TKIs such as gefitinib, erlotinib, and afatinib.^7^ AURA‐3 reported 10.1 months of PFS on osimertinib and 4.4 months of PFS on platinum‐based pemetrexed therapy, with results comparable to those of RESET, 11.9 months in group A and 5.1 months in group B.

RWD may differ from RCT data for several reasons. RCTs traditionally require strict criteria for study entry, but this would guarantee unbiased distribution of confounding factors, support causality, and provide strong internal validity; thus, evidence from the RCTs has been considered the gold standard. However, because patients with poor performance status, presence of brain metastasis, advanced age, and comorbidities are seldom included, RCTs may suffer from the loss of clinical diversity. Therefore, the importance of RWD in clinical practice has become apparent. RWD can provide supplemental data and additional understanding on top of that from RCTs.^16^ In particular, concordance between RWD and RCT data could establish the best approach for managing patients.

However, in terms of sequential therapy with afatinib and osimertinib, only a small number of studies have investigated the clinical characteristics and outcomes in patients with EGFR‐mutated NSCLC. In the real‐world GioTag study,^8^ when using sequential treatment of afatinib and osimertinib, encouraging TOT results were obtained, especially in Asian populations and Del19‐positive NSCLC patients. The median TOT was 27.6 months in all patients, 30.3 months in Del19‐positive NSCLC patients, and 46.7 months in Asian populations.^8^ Updated data on the GioTag study showed that the median OS was 41.3 months and the TTF was 28.1 months.^17^ In Del19‐positive NSCLC patients, the OS was 45.7 months and the TTF was 30.6 months.^17^ Although an advantage of the GioTag study is its involvement of a variety of ethnic groups in several countries, it only included 50 Asian patients. During first‐ and second‐line treatments, the overall TOT in all patients within RESET was 25.9 months. The median TOT in group A patients within RESET was estimated to be 35.4 months, which is slightly shorter than that of Asian patients within GioTag, 46.7 months.^8^ However, given that only a small number of Asian patients were included in GioTag, the proportion of elderly patients (≥65 years) was higher in RESET (44.1 vs. 34.8%), and RESET included a higher percentage of patients with baseline brain metastasis (43.8 vs. 10.3%),^8^ the results of RESET are encouraging. The RWD from the RESET study is further supported by another multicenter retrospective study in Japanese patients.^18^ In this study, patients sequentially treated with afatinib and osimertinib showed better ORR and DCR than patients treated with other first‐generation TKIs (i.e., gefitinib and erlotinib). The above observational studies using RWD highlight the efficacy of sequential treatment with afatinib and osimertinib.

Several RWD trials have estimated the efficacy of afatinib as a first‐line treatment option in patients with EGFR‐mutated NSCLC. The estimated TOT‐1 in RESET (15.7 months) is similar to previously reported results from other real‐world studies: TOT of 14.0 months for Asian populations in the GioTag study,^8^ TTF of 13.1 months in the Japanese population,^10^ and TTF of 13.6 months and PFS of 12.4 months in a Taiwanese group.^13^ In contrast, in the Korean population, Kim et al. reported a longer PFS for first‐line afatinib treatment (19.1 months) than in our study. This difference might be attributable to the different characteristics of the study subjects. Compared to the same ethnic group in the study by Kim et al., which analyzed 165 Koreans diagnosed with NSCLC, the subjects in our study were older (median age: 61.5 years vs. 57 years) and presented with a slightly higher percentage of brain metastasis (43.8 vs. 40.6%).^9^

RESET assessed several factors affecting TOT‐1 using a multivariate Cox PH model. Poor performance status (ECOG PS ≥2 vs. 0–1), advanced tumor stage (AJCC 4B vs. 3), tissue type other than adenocarcinoma, liver metastasis, and no afatinib dose adjustment were shown to be related to decreased TOT‐1. Interestingly, afatinib dose adjustment was associated with better outcomes for TOT. It is not clear whether TOT was better due to dose reductions or whether TOT was worse due to dose maintenance, but a study that investigated the effect of dose adjustment on survival outcomes in patients with EGFR‐mutated NSCLC reported that patients who received dose reductions experienced higher ORRs.^19^ Another RWD study showed that dose adjustment reduced the number and intensity of several side effects, emphasizing that tailored dose modification could help treatment optimization and improve survival outcomes.^20^ In addition, a post hoc analysis of LUX‐Lung 3 and 6 trials showed that dose reduction was more common in female, old‐age, low‐weight, and Asian‐Japanese patients.^21^ Dose reduction led to fewer adverse events and less treatment discontinuation.

Notably, the TOT‐1 in group A patients was greater than that in group B patients. All patients in group A presented T790M mutation after afatinib, while patients in group B did not. This result is consistent with another study showing that OS was significantly longer in patients with the T790M mutation than in those without it.^22^ Tanaka et al. reported that PFS was significantly longer in patients who developed the T790M mutation after the onset of first‐line afatinib therapy than in those who did not develop the mutation.^23^ Although the mechanism of resistance to EGFR‐TKIs might be heterogeneous, the slow growth rate of T790M‐harboring cells could partially explain this observation.^24^ These findings might account for the better survival outcomes in patients who developed the T790M mutation after first‐line afatinib treatment, indicating the potential association between a longer treatment period and the development of the mutation.

Several EGFR‐TKIs have been developed to address the problem of EGFR mutations following therapy. Currently, the standard treatment option for patients with EGFR‐mutated advanced‐stage NSCLC is an EGFR‐TKI. Considering its superior efficacy in terms of PFS, OS, central nervous system activity, and adverse events, clinicians prefer osimertinib as first‐line therapy over other EGFR‐TKIs.^25^ Despite these benefits, inevitably acquired mutations during osimertinib therapy, such as the C797S mutation,^26^ as well as the interpatient, intratumoral, and intertumoral heterogeneity of NSCLC,^27^ complicate the optimal therapeutic determination of EGFR‐TKI sequence. The optimal sequence of treatment remains controversial.^15^ In particular, in the National Health Insurance of South Korea, osimertinib is only approved for second‐line treatment after the failure of other first‐line EGFR‐TKI treatments. Therefore, sequential treatment of first‐ or second‐generation EGFR‐TKIs with the third‐generation EGFR‐TKI, osimertinib, is a possible alternative.

The RESET study has limitations. First, the main limitation resulted from RESET's retrospective nature. Selection bias or misclassification existed. To mitigate this problem, subgroup analyses were performed to identify any potential factors significantly affecting survival outcomes. The results of multicenter hospital‐based surveillance from RESET could provide insight into the universality of the efficacy of sequential treatment with afatinib and osimertinib. Second, TOT‐2 was shorter than TOT‐1, which is opposite to the finding in GioTag. TOT‐1 in GioTag might have been rather short due to inclusion criteria and drug availability. And also, this may have originated from the short observation period of osimertinib treatment in RESET, in which survival data were not matured at the time of analysis; further data collection and analysis may be warranted. Third, because 413 patients were excluded, it was not feasible to obtain more detailed information, such as the percentage of patients who received second‐line treatment or the frequency of T790M development. In the future, we are planning to also collect data in group A and B patients and in excluded patients who received first‐line afatinib treatment. Despite these limitations, to the best of our knowledge, RESET is the first multicenter study in South Korea based on real‐world experience, and its applicability to real clinical practice, especially for Asian populations, could allow better patient management and improved survival outcomes in patients with EGFR‐mutated advanced NSCLC.

## 5 CONCLUSIONS

Real‐world experience of sequential treatment of afatinib and osimertinib (RESET) is the first multicenter study in South Korea in patients with EGFR‐mutated advanced NSCLC, comprehensively comparing the RWD of sequential treatment with afatinib and osimertinib to the RWD of other second‐line treatments. Osimertinib after first‐line afatinib treatment was superior to other regimens as second‐line treatments in terms of TOT, OS, ORR, and DCR, especially in patients presenting T790M mutation after afatinib. Our results show the feasibility of sequential treatment with afatinib and osimertinib in patients with EGFR‐mutated advanced NSCLC, maximizing sustained clinical benefit and minimizing exposure to chemotherapy.

## ETHICAL APPROVAL STATEMENT

The study and protocol were approved by the Institutional Review Board of the Kosin University Gospel Hospital (KUGH no. 2019‐07‐038). The study was conducted following the Declaration of Helsinki. All procedures were performed in accordance with the relevant guidelines and regulations.

## CONFLICT OF INTEREST

no conflict of interest.

## AUTHOR CONTRIBUTIONS

**T Kim**: Writing ‐ original draft, Methodology, Software, Formal analysis, Data Curation, Visualization. **TW Jang**: Conceptualization, Funding acquisition, Writing ‐ review & editing, Supervision, Project administration. **All authors**: Investigation, Validation. All authors discussed the results and approved the final version of the manuscript.

## Supporting information

Fig S1

Fig S2

Fig S3

Table S1‐S6

## Data Availability

The data that support the findings of this study are available from the corresponding author upon reasonable request.
