# Supplementary figures and images for: Sequential treatment of afatinib and osimertinib or other regimens in patients with advanced non‐small‐cell lung cancer harboring EGFR mutations: Results from a real‐world study in South Korea
Source: Cancer Med. 2021 Jul 13;10(17):5809–22. doi: 10.1002/cam4.4127 (PMC8419762; doi:10.1002/cam4.4127)

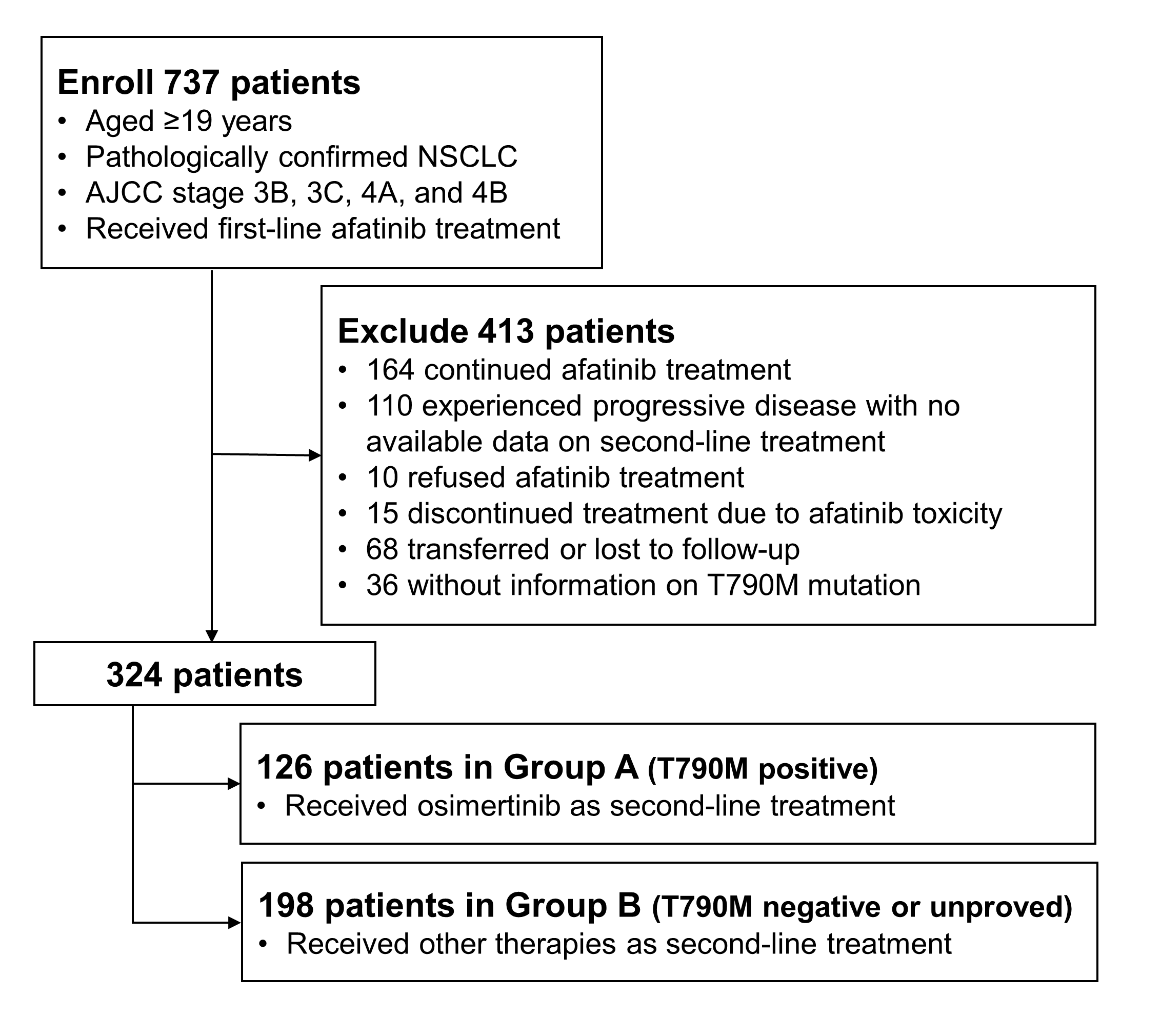

Supplement: Supplementary file 1 — Fig S1 [file CAM4-10-5809-s001.tif]

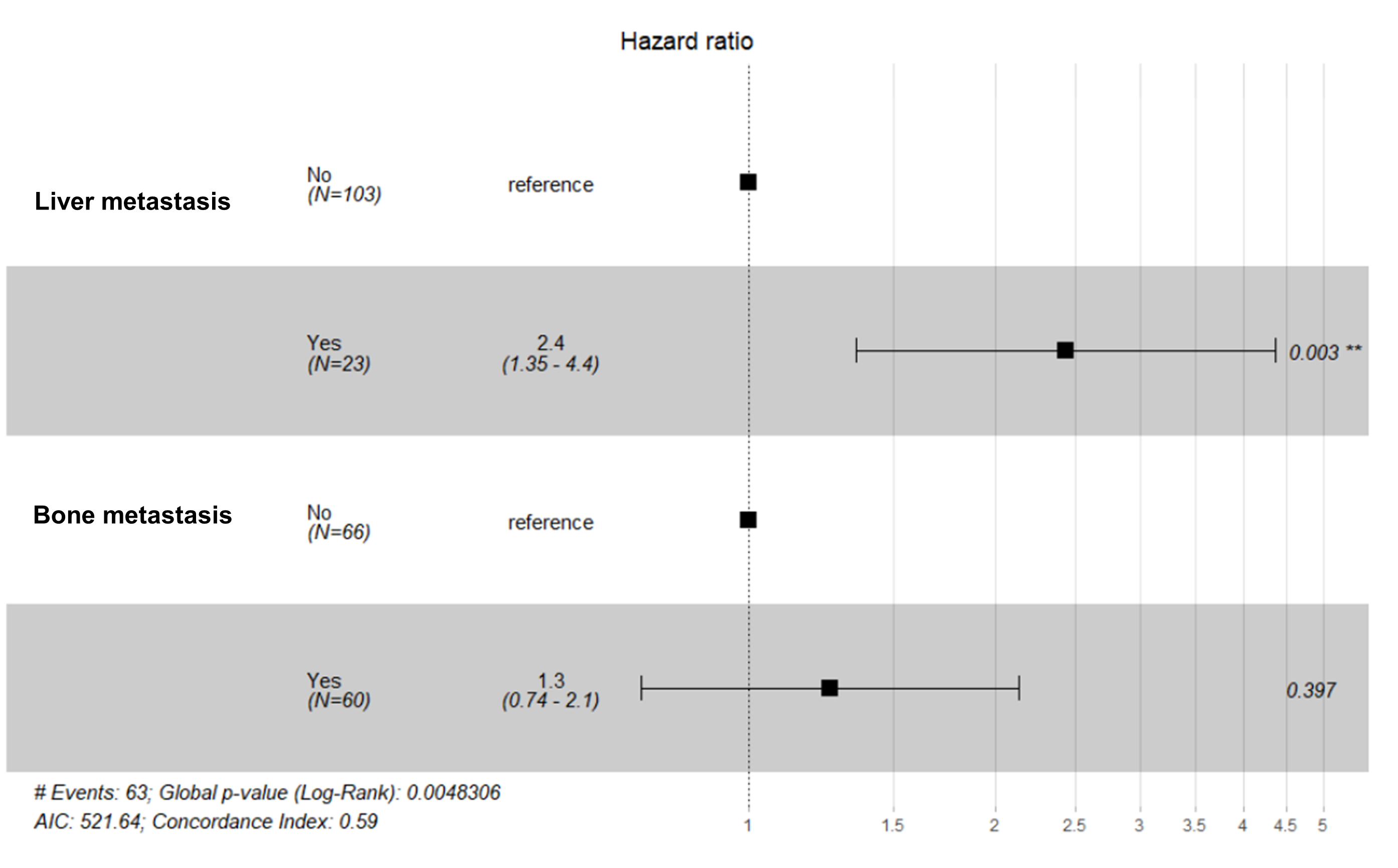

Supplement: Supplementary file 2 — Fig S2 [file CAM4-10-5809-s004.tif]

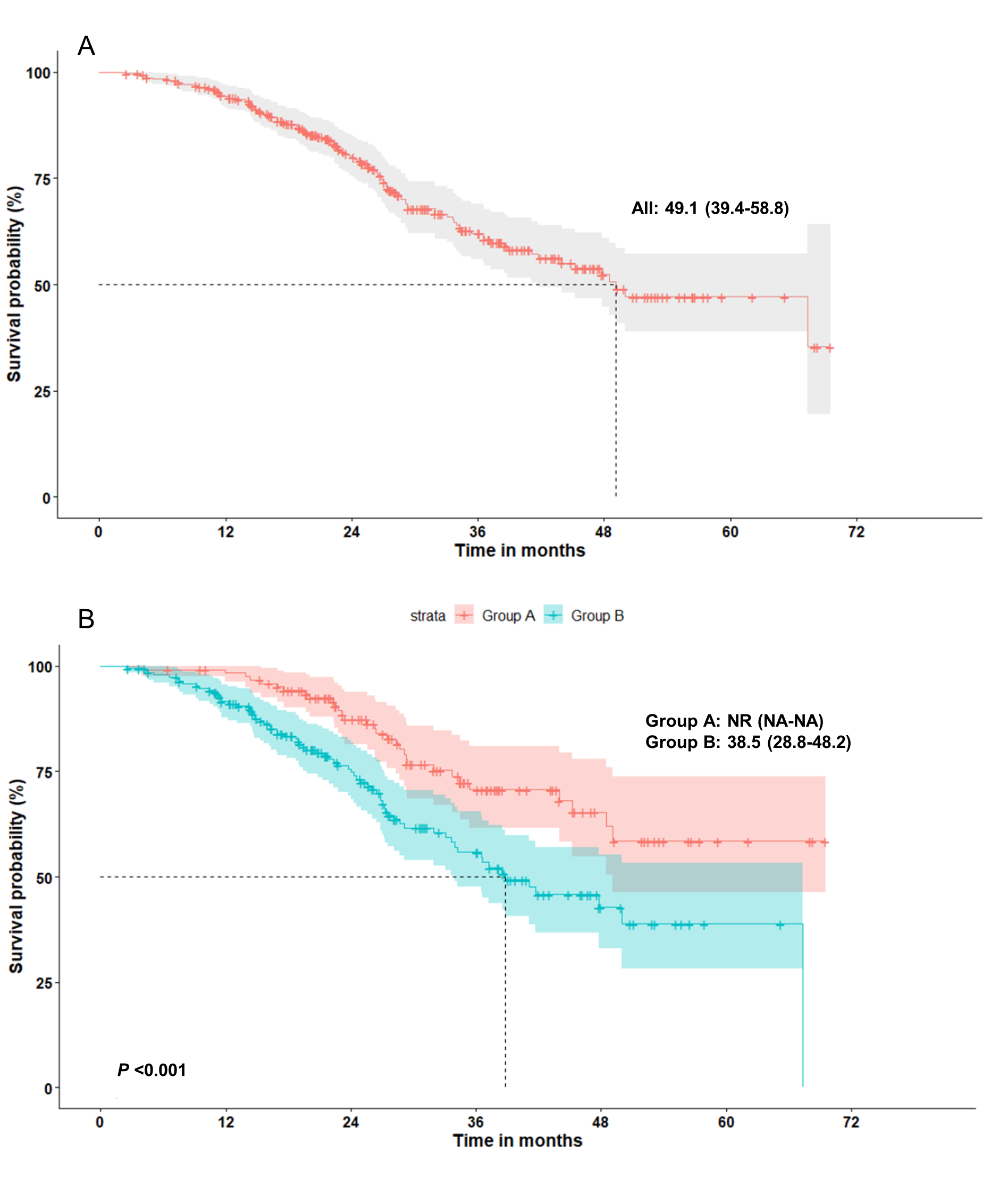

Supplement: Supplementary file 3 — Fig S3 [file CAM4-10-5809-s003.tif]
