# Supplementary material for: Sequential treatment of afatinib and osimertinib or other regimens in patients with advanced non‐small‐cell lung cancer harboring EGFR mutations: Results from a real‐world study in South Korea
Source: Cancer Med. 2021 Jul 13;10(17):5809–22. doi: 10.1002/cam4.4127 (PMC8419762; doi:10.1002/cam4.4127)
Supplement: Supplementary file 4 — Table S1‐S6 [file CAM4-10-5809-s002.docx]

**Table S1.** Objective response rate in patients who received first-line afatinib treatment

|  | Total (n=311/324) | *P* | Group A (n=124/126) | *P* | Group B (n=187/198) | *P* |
| --- | --- | --- | --- | --- | --- | --- |
| Overall | 69.1% |  | 75.0% |  | 65.2% |  |
| Age |  | 0.088 |  | 0.526 |  | 0.093 |
| <65 | 73.3% |  | 77.0% |  | 70.6% |  |
| ≥65 | 63.7% |  | 72.0% |  | 55.5% |  |
| Sex |  | 0.542 |  | 1.000 |  | 0.449 |
| Men | 70.6% |  | 75.0% |  | 67.6% |  |
| Women | 67.4% |  | 75.0% |  | 62.4% |  |
| ECOG PS |  | 0.169 |  | 1.000 |  | 0.111 |
| 0 or 1 | 68.8% |  | 72.7% |  | 66.5% |  |
| ≥2 | 54.5% |  | 80.0% |  | 47.1% |  |
| Stage§ |  | 0.914 |  | 0.078 |  | 0.056 |
| 3 and 4A | 69.3% |  | 67.8% |  | 70.0% |  |
| 4B | 68.7% |  | 81.5% |  | 56.1% |  |
| Smoking |  | 0.978 |  | 0.103 |  | 0.247 |
| Never | 68.4% |  | 73.3% |  | 65.2% |  |
| Former | 69.7% |  | 70.3% |  | 69.2% |  |
| Current | 69.0% |  | 100% |  | 47.1% |  |
| Tissue type |  | 0.444 |  | 0.572 |  | 1.000 |
| Adenocarcinoma | 68.8% |  | 74.4% |  | 65.0% |  |
| Others¶ | 85.7% |  | 100% |  | 75.0% |  |
| EGFR |  | 0.011 |  | 0.322 |  | 0.019 |
| Del19 | 75.9% |  | 78.9% |  | 73.5% |  |
| L858R | 62.8% |  | 65.7% |  | 61.0% |  |
| Others† | 55.8% |  | 76.9% |  | 46.7% |  |
| # of metastatic organs | | 0.625 |  | 0.816 |  | 0.858 |
| 0-1 | 66.4% |  | 72.0% |  | 63.5% |  |
| 2-3 | 71.6% |  | 77.2% |  | 67.5% |  |
| 4 or more | 71.0% |  | 76.5% |  | 64.3% |  |
| Adrenal gland meta. |  | 0.464 |  | 0.729 |  | 0.144 |
| Yes | 62.5% |  | 81.8% |  | 46.2% |  |
| No | 69.7% |  | 74.3% |  | 66.7% |  |
| Liver meta. |  | 0.480 |  | 0.786 |  | 0.636 |
| Yes | 73.8% |  | 77.3% |  | 70.0% |  |
| No | 68.4% |  | 74.5% |  | 64.7% |  |
| Bone meta. |  | 0.679 |  | 0.917 |  | 0.658 |
| Yes | 93.3% |  | 74.6% |  | 67.1% |  |
| No | 68.2% |  | 75.4% |  | 64.0% |  |
| Brain meta. |  | 0.883 |  | 0.530 |  | 0. 539 |
| Yes | 69.6% |  | 72.2% |  | 67.9% |  |
| No | 68.8% |  | 77.1% |  | 63.1% |  |
| Type of brain meta. |  | 0.491 |  | 1.000 |  | 0.537 |
| Single parenchymal | 76.2% |  | 75.0% |  | 76.9% |  |
| Multiple +/- seeding | 80.3% |  | 71.7% |  | 66.7% |  |
| New lesion or aggravation of brain meta. | | 0.360 |  | 0.600 |  | 0.741 |
| Yes | 65.1% |  | 70.8% |  | 62.7% |  |
| No | 70.5% |  | 76.0% |  | 66.1% |  |
| Dose adj. for afatinib |  | 0.306 |  | 0.822 |  | 0.362 |
| Yes | 71.4% |  | 75.6% |  | 68.1% |  |
| No | 65.8% |  | 73.7% |  | 61.6% |  |

Data are presented as numbers (percentages), unless otherwise stated.

Patients in group A received sequential treatment of afatinib and osimertinib, while patients in group B received other therapies following first-line afatinib treatment.

**§** Tumor stage was classified based on 8^th^ edition of the American Joint Committee on Cancer staging system.

¶ Other tissue types included squamous cell carcinoma in 2 patients, adenosquamous cell carcinoma in 2 patients, and non-small cell lung cancer in 3 patients.

† Patients not presenting with EGFR Del19 and L858R mutations, including de novo T790M mutation, are classified as the “Others” group.

ECOG PS, Eastern Cooperative Oncology Group performance status; EGFR, epidermal growth factor receptor; Del19, deletion 19; meta., metastasis; adj., adjustment

.

**Table S2.** Disease control rate in patients who received first-line afatinib treatment

|  | Total (n=311/324) | *P* | Group A (n=124/126) | *P* | Group B (n=187/198) | *P* |
| --- | --- | --- | --- | --- | --- | --- |
| Overall | 93.2% |  | 97.6% |  | 90.4% |  |
| Age |  | 0.687 |  | 1.000 |  | 0.684 |
| <65 | 93.8% |  | 97.3% |  | 91.2% |  |
| ≥65 | 92.6% |  | 98.0% |  | 89.4% |  |
| Sex |  | 0.828 |  | 0.251 |  | 0.365 |
| Men | 93.5% |  | 95.6% |  | 92.2% |  |
| Women | 92.9% |  | 100% |  | 88.2% |  |
| ECOG PS |  | 1.000 |  | 1.000 |  | 1.000 |
| 0 or 1 | 92.4% |  | 97.0% |  | 89.6% |  |
| ≥2 | 95.5% |  | 100% |  | 94.1% |  |
| Stage§ |  | 0.391 |  | 0.105 |  | 0.798 |
| 3 and 4A | 92.2% |  | 94.9% |  | 90.8% |  |
| 4B | 94.7% |  | 100% |  | 89.4% |  |
| Smoking |  | 0.892 |  | 0.183 |  | 0.823 |
| Never | 93.7% |  | 100% |  | 89.6% |  |
| Former | 92.1% |  | 91.9% |  | 92.3% |  |
| Current | 93.1% |  | 100% |  | 88.2% |  |
| Tissue type |  | 1.000 |  | 1.000 |  | 1.000 |
| Adenocarcinoma | 93.1% |  | 97.5% |  | 90.2% |  |
| Others¶ | 100% |  | 100% |  | 100% |  |
| EGFR |  | 0.021 |  | 0.377 |  | 0.079 |
| Del19 | 96.6% |  | 98.7% |  | 94.9% |  |
| L858R | 90.4% |  | 97.1% |  | 86.4% |  |
| Others† | 86.0% |  | 92.3% |  | 83.3% |  |
| # of metastatic organs | | 0.538 |  | 0.103 |  | 0.924 |
| 0-1 | 91.8% |  | 94.0% |  | 90.6% |  |
| 2-3 | 94.0% |  | 100% |  | 89.6% |  |
| 4 or more | 96.8% |  | 100% |  | 92.9% |  |
| Adrenal gland meta. |  | 0.213 |  | 1.000 |  | 0.116 |
| Yes | 87.5% |  | 100% |  | 76.9% |  |
| No | 93.7% |  | 97.3% |  | 91.4% |  |
| Liver meta. |  | 0.751 |  | 1.000 |  | 1.000 |
| Yes | 95.2% |  | 100% |  | 90.0% |  |
| No | 92.9% |  | 97.1% |  | 90.4% |  |
| Bone meta. |  | 0.611 |  | 0.246 |  | 0.730 |
| Yes | 94.1% |  | 100% |  | 89.5% |  |
| No | 92.6% |  | 95.4% |  | 91.0% |  |
| Brain meta. |  | 0.131 |  | 0.256 |  | 0.299 |
| Yes | 95.7% |  | 100% |  | 92.9% |  |
| No | 91.3% |  | 95.7% |  | 88.3% |  |
| Type of brain meta. | | 0.590 |  | NA |  | 0.583 |
| Single parenchymal | 100% |  | 100% |  | 100% |  |
| Multiple +/- seeding | 94.8% |  | 100% |  | 91.3% |  |
| New lesion or aggravation of brain meta. | | 0.408 |  | 1.000 |  | 0.362 |
| Yes | 95.2% |  | 100% |  | 93.2% |  |
| No | 92.5% |  | 97.0% |  | 89.0% |  |
| Dose adj. for afatinib | | 0.010 |  | 1.000 |  | 0.012 |
| Yes | 96.0% |  | 97.7% |  | 94.7% |  |
| No | 88.3% |  | 97.4% |  | 83.6% |  |

Data are presented as numbers (percentages), unless otherwise stated.

Patients in group A received sequential treatment with afatinib and osimertinib, while patients in group B received other therapies following first-line afatinib treatment.

**§** Tumor stage was classified based on 8^th^ edition of the American Joint Committee on Cancer staging system.

¶ Other tissue types included squamous cell carcinoma in 2 patients, adenosquamous cell carcinoma in 2 patients, and non-small cell lung cancer in 3 patients.

† Patients not presenting with EGFR Del19 and L858R mutations, including de novo T790M mutation, are classified as the “Others” group.

ECOG PS, Eastern Cooperative Oncology Group performance status; EGFR, epidermal growth factor receptor; Del19, deletion 19; meta., metastasis; adj., adjustment

**Table S3.** Objective response rate in patients who received second-line treatment

|  | Group A (n=112/126) | *P* | Group B (n=161/198) | *P* |
| --- | --- | --- | --- | --- |
| Overall | 40.2% |  | 20.5% |  |
| Age |  | 0.558 |  | 0.869 |
| <65 | 42.6% |  | 20.0% |  |
| ≥65 | 36.4% |  | 21.1% |  |
| Sex |  | 0.724 |  | 0.569 |
| Men | 38.7% |  | 18.9% |  |
| Women | 42.0% |  | 22.5% |  |
| ECOG PS |  | 0.645 |  | 0.530 |
| 0 or 1 | 41.6% |  | 21.0% |  |
| ≥2 | 20.0% |  | 11.1% |  |
| Stage§ |  | 0.264 |  | 0.738 |
| 3 and 4A | 34.6% |  | 20.8% |  |
| 4B | 45.0% |  | 18.5% |  |
| Smoking |  | 0.705 |  | 0.983 |
| Never | 37.7% |  | 20.2% |  |
| Former | 41.9% |  | 20.9% |  |
| Current | 50.0% |  | 18.8% |  |
| Tissue type |  | 0.150 |  | 0.582 |
| Adenocarcinoma | 41.3% |  | 21.0% |  |
| Others¶ | 0.0% |  | 0.0% |  |
| EGFR |  | 0.964 |  | 0.284 |
| Del19 | 40.6% |  | 15.7% |  |
| L858R | 40.6% |  | 26.5% |  |
| Others† | 36.4% |  | 24.1% |  |
| # of metastatic organs | | 0.806 |  | 0.146 |
| 0-1 | 42.9% |  | 24.1% |  |
| 2-3 | 37.0% |  | 19.4% |  |
| 4 or more | 43.8% |  | 0.0% |  |
| Adrenal gland meta. |  | 0.507 |  | 0.895 |
| Yes | 50.0% |  | 22.2% |  |
| No | 39.2% |  | 20.4% |  |
| Liver meta. |  | 0.684 |  | 0.079 |
| Yes | 36.4% |  | 5.0% |  |
| No | 41.1% |  | 22.7% |  |
| Bone meta. |  | 0.335 |  | 0.422 |
| Yes | 35.7% |  | 16.7% |  |
| No | 44.6% |  | 22.8% |  |
| Brain meta. |  | 0.464 |  | 0.515 |
| Yes | 36.5% |  | 22.9% |  |
| No | 43.3% |  | 18.7% |  |
| Type of brain meta. |  | 0.694 |  | 0.450 |
| Single parenchymal | 25.0% |  | 33.3% |  |
| Multiple +/- seeding | 38.6% |  | 20.7% |  |
| New lesion or aggravation of brain meta. | | 0.440 |  | <0.001 |
| Yes | 33.3% |  | 3.8% |  |
| No | 42.0% |  | 29.0% |  |
| Dose adj. for afatinib |  | 0.825 |  | 0.012 |
| Yes | 39.5% |  | 13.5% |  |
| No | 41.7% |  | 29.7% |  |

Data are presented as numbers (percentages), unless otherwise stated.

Patients in group A received sequential treatment with afatinib and osimertinib, while patients in group B received other therapies following first-line afatinib treatment.

**§** Tumor stage was classified based on 8^th^ edition of the American Joint Committee on Cancer staging system.

¶ Other tissue types included squamous cell carcinoma in 2 patients, adenosquamous cell carcinoma in 2 patients, and non-small cell lung cancer in 3 patients.

† Patients not presenting with EGFR Del19 and L858R mutations, including de novo T790M mutation, are classified as the “Others” group.

ECOG PS, Eastern Cooperative Oncology Group performance status; EGFR, epidermal growth factor receptor; Del19, deletion 19; meta., metastasis; adj., adjustment

**Table S4.** Disease control rate in patients who received second-line treatment

|  | Group A (n=112/126) | *P* | Group B (n=161/198) | *P* |
| --- | --- | --- | --- | --- |
| Overall | 94.6% |  | 75.2% |  |
| Age |  | 0.678 |  | 0.292 |
| <65 | 95.6% |  | 71.8% |  |
| ≥65 | 93.2% |  | 78.9% |  |
| Sex |  | 0.222 |  | 0.814 |
| Men | 91.9% |  | 74.4% |  |
| Women | 98.0% |  | 76.1% |  |
| ECOG PS |  | 1.000 |  | 0.393 |
| 0 or 1 | 94.4% |  | 76.1% |  |
| ≥2 | 100% |  | 66.7% |  |
| Stage§ |  | 0.414 |  | 0.562 |
| 3 and 4A | 92.3% |  | 73.6% |  |
| 4B | 96.7% |  | 77.8% |  |
| Smoking |  | 0.294 |  | 0.317 |
| Never | 92.8% |  | 78.8% |  |
| Former | 100% |  | 72.1% |  |
| Current | 91.7% |  | 62.5% |  |
| Tissue type |  | 1.000 |  | 1.000 |
| Adenocarcinoma | 94.5% |  | 75.2% |  |
| Others¶ | 100% |  | 75.0% |  |
| EGFR |  | 0.725 |  | 0.405 |
| Del19 | 94.2% |  | 79.5% |  |
| L858R | 96.9% |  | 71.4% |  |
| Others† | 90.9% |  | 69.0% |  |
| # of metastatic organs | | 0.134 |  | 0.005 |
| 0-1 | 100% |  | 80.5% |  |
| 2-3 | 90.7% |  | 75.8% |  |
| 4 or more | 93.8% |  | 33.3% |  |
| Adrenal gland meta. |  | 1.000 |  | 0.228 |
| Yes | 100% |  | 55.6% |  |
| No | 94.1% |  | 76.3% |  |
| Liver meta. |  | 0.336 |  | 0.001 |
| Yes | 90.9% |  | 45.0% |  |
| No | 95.6% |  | 79.4% |  |
| Bone meta. |  | 0.679 |  | 0.732 |
| Yes | 92.9% |  | 76.7% |  |
| No | 96.4% |  | 74.3% |  |
| Brain meta. |  | 1.000 |  | 0.337 |
| Yes | 94.2% |  | 71.4% |  |
| No | 95.0% |  | 68.4% |  |
| Type of brain meta. |  | 1.000 |  | 0.727 |
| Single parenchymal | 100% |  | 66.7% |  |
| Multiple +/- seeding | 93.2% |  | 72.4% |  |
| New lesion or aggravation of brain meta. | | 1.000 |  | 0.771 |
| Yes | 95.8% |  | 73.6% |  |
| No | 94.3% |  | 75.7% |  |
| Dose adj. for afatinib |  | 0.384 |  | 1.000 |
| Yes | 96.1% |  | 75.0% |  |
| No | 91.7% |  | 75.0% |  |

Data are presented as numbers (percentages), unless otherwise stated.

Patients in group A received sequential treatment with afatinib and osimertinib, while patients in group B received other therapies following first-line afatinib treatment.

**§** Tumor stage was classified based on 8^th^ edition of the American Joint Committee on Cancer staging system.

¶ Other tissue types included squamous cell carcinoma in 2 patients, adenosquamous cell carcinoma in 2 patients, and non-small cell lung cancer in 3 patients.

† Patients not presenting with EGFR Del19 and L858R mutations, including de novo T790M mutation, are classified as the “Others” group.

ECOG PS, Eastern Cooperative Oncology Group performance status; EGFR, epidermal growth factor receptor; Del19, deletion 19; meta., metastasis; adj., adjustment

**Table S5.** Median overall survival with 2-year and 3-year survival rates in all patients

|  | Total (n=324) | | | |
| --- | --- | --- | --- | --- |
|  | **2-y** | **3-y** | **OS (95% CI)** | *P* |
| Overall | 78.1% | 63.5% | 49.1 (39.4-58.8) |  |
| Age |  |  |  | 0.205 |
| <65 | 79.7% | 63.7% | 44.0 (35.2-52.8) |  |
| ≥65 | 76.4% | 64.0% | 52.9 (46.4-59.4) |  |
| Sex |  |  |  | 0.267 |
| Men | 76.4% | 59.8% | 52.9 (34.6-71.2) |  |
| Women | 79.8% | 67.5% | 48.5 (41.1-55.9) |  |
| ECOG PS |  |  |  | 0.045 |
| 0 or 1 | 80.3% | 66.1% | 50.0 (41.2-58.8) |  |
| ≥2 | 61.4% | 38.3% | 28.7 (17.6-39.8) |  |
| Stage§ |  |  |  | 0.013 |
| 3 and 4A | 81.9% | 70.0% | 67.3 (42.2, 92,4) |  |
| 4B | 71.9% | 51.9% | 36.6 (26.8-46.4) |  |
| Smoking |  |  |  | 0.534 |
| Never | 80.1% | 65.4% | 50.0 (38.8-61.2) |  |
| Former | 74.4% | 61.1% | 47.8 (37.3-58.3) |  |
| Current | 75.5% | 58.6% | 45.2 (NA-NA) |  |
| Tissue type |  |  |  | 0.070 |
| Adenocarcinoma | 78.4% | 63.7% | 49.1 (39.1-59.1) |  |
| Others¶ | 61.1% | 61.1% | 44.0 (NA-NA) |  |
| EGFR |  |  |  | 0.020 |
| Del19 | 84.9% | 67.5% | 67.3 (44.4-90.2) |  |
| L858R | 75.3% | 52.7% | 36.6 (28.1-45.1) |  |
| Others† | 73.6% | 56.8% | NR (NA-NA) |  |
| # of metastatic organs |  |  |  | <0.001 |
| 0-1 | 85.1% | 74.8% | NR (NA-NA) |  |
| 2-3 | 70.3% | 50.5% | 36.3 (27.1-46.1) |  |
| 4 or more | 66.0% | 38.3% | 28.7 (21.2-36.2) |  |
| Adrenal gland meta. |  |  |  | 0.588 |
| Yes | 75.0% | 54.2% | 48.5 (14.2-82.8) |  |
| No | 78.3% | 64.2% | 49.1 (39.0-59.2) |  |
| Liver meta. |  |  |  | <0.001 |
| Yes | 60.6% | 42.1% | 28.7 (21.1-36.2) |  |
| No | 80.0% | 65.9% | 52.9 (43.3-62.5) |  |
| Bone meta. |  |  |  | <0.001 |
| Yes | 70.7% | 46.2% | 29.4 (24.8-34.0) |  |
| No | 83.3% | 74.8% | NR (NA-NA) |  |
| Brain meta. |  |  |  | 0.031 |
| Yes | 72.4% | 51.3% | 37.2 (25.1-49.3) |  |
| No | 81.8% | 71.1% | 67.3 (39.9-95.0) |  |
| Type of brain meta. |  |  |  | 0.311 |
| Single parenchymal | 69.3% | 56.0% | 37.2 (21.4-53.0) |  |
| Multiple +/- seeding | 80.6% | 52.3% | 36.6 (21.6-51.6) |  |
| New lesion or aggravation of brain meta. | | |  | 0.003 |
| Yes | 69.4% | 49.0% | 34.0 (24.0-44.0) |  |
| No | 80.8% | 68.1% | 67.3 (41.5-93.1)) |  |
| Dose adj. for afatinib |  |  |  | 0.286 |
| Yes | 81.0% | 65.5% | 50.0 (NA-NA) |  |
| No | 80.5% | 56.1% | 47.8 (31.8-63.8) |  |

Data are presented as percentages for survival rates and months (95% confidence intervals) for overall survival, unless otherwise stated.

Patients in group A received sequential treatment with afatinib and osimertinib, while patients in group B received other therapies following first-line afatinib treatment.

**§** Tumor stage was classified based on 8^th^ edition of the American Joint Committee on Cancer staging system.

¶ Other tissue types included squamous cell carcinoma in 2 patients, adenosquamous cell carcinoma in 2 patients, and non-small cell lung cancer in 3 patients.

† Patients not presenting with EGFR Del19 and L858R mutations, including de novo T790M mutation, are classified as the “Others” group.

ECOG PS, Eastern Cooperative Oncology Group performance status; EGFR, epidermal growth factor receptor; Del19, deletion 19; meta., metastasis; adj., adjustment; CI, confidence interval; OS, overall survival; NR, not reached; NA, not-available.

**Table S6.** Respective median overall survival with 2-year and 3-year survival rates in group A and B patients

|  | Group A (n=126) | | | | Group B (n=198) | | | |
| --- | --- | --- | --- | --- | --- | --- | --- | --- |
|  | **2-year** | **3-year** | **OS (95% CI)** | *P* | **2-year** | **3-year** | **OS (95% CI)** | *P* |
| Overall | 86.0% | 69.3% | NR (NA-NA) |  | 75.9% | 55.3% | 38.5 (28.8-48.2) |  |
| Age |  |  |  | 0.151 |  |  |  | 0.590 |
| <65 | 83.4% | 63.5% | NR (NA-NA) |  | 73.4% | 53.4% | 37.2 (27.1-47.3) |  |
| ≥65 | 89.8% | 78.7% | NR (NA-NA) |  | 79.0% | 58.3% | 47.8 (27.9-67.7) |  |
| Sex |  |  |  | 0.357 |  |  |  | 0.031 |
| Men | 87.0% | 71.4% | NR (NA-NA) |  | 69.2% | 46.4% | 31.9 (23.6-40.2) |  |
| Women | 84.9% | 67.4% | 49.1 (NA-NA) |  | 83.5% | 65.3% | 41.7 (30.6-52.8) |  |
| ECOG PS |  |  |  | 0.004 |  |  |  | 0.513 |
| 0 or 1 | 90.0% | 70.1% | NR (NA-NA) |  | 76.1% | 58.1% | 41.1 (31.5-50.7) |  |
| ≥2 | 30.0% | 30.0% | 22.3 (9.8-34.8) |  | 80.4% | 42.9% | 33.6 (23.4-43.8) |  |
| Stage§ |  |  |  | 0.006 |  |  |  | 0.091 |
| 3 and 4A | 89.9% | 78.0% | NR (NA-NA) |  | 80.3% | 60.4% | 47.8 (35.8-59.8) |  |
| 4B | 82.4% | 58.9% | 44.0 (30.6-57.4) |  | 64.9% | 42.0% | 27.5 (20.1-34.9) |  |
| Smoking |  |  |  | 0.384 |  |  |  | 0.039 |
| Never | 85.3% | 68.4% | NR (NA-NA) |  | 81.6% | 59.3% | 41.1 (29.6-52.6) |  |
| Former | 81.9% | 66.6% | 49.1 (NA-NA) |  | 71.7% | 54.1% | 38.5 (23.9-53.1) |  |
| Current | 100% | 81.5% | NR (NA-NA) |  | 53.5% | 32.1% | 26.5 (7.9-45.1) |  |
| Tissue type |  |  |  | 0.126 |  |  |  | 0.160 |
| Adenocarcinoma | 86.4% | 69.5% | NR (NA-NA) |  | 76.4% | 55.5% | 38.8 (28.9-48.7) |  |
| Others¶ | 66.7% | 66.7% | 44.0 (NA-NA) |  | 50.0% | NA | 9.4 (NA-NA) |  |
| EGFR |  |  |  | 0.236 |  |  |  | 0.093 |
| Del19 | 90.0% | 76.3% | NR (NA-NA) |  | 80.1% | 59.2% | 50.0 (41.5-58.5) |  |
| L858R | 80.8% | 56.3% | 45.2 (NA-NA) |  | 70.9% | 50.4% | 36.5 (26.8-46.2) |  |
| Others† | 74.7% | 65.3% | NR (NA-NA) |  | 72.7% | 49.5% | 34.2 (NA-NA) |  |
| # of metastatic organs |  |  |  | <0.001 |  |  |  | 0.007 |
| 0-1 | 95.1% | 83.1% | NR (NA-NA) |  | 84.1% | 66.9% | 50.0 (36.5-63.5) |  |
| 2-3 | 83.3% | 63.5% | 45.2 (34.8-55.6) |  | 64.2% | 40.6% | 27.5 (25.0-30.0) |  |
| 4 or more | 65.6% | 41.0% | 28.7 (19.9-37.5) |  | 76.2% | 38.1% | 25.6 (22.9-28.3) |  |
| Adrenal gland meta. |  |  |  | 0.790 |  |  |  | 0.354 |
| Yes | 76.2% | 76.2% | NR (NA-NA) |  | 66.7% | 33.3% | 26.8 (13.9-40.0) |  |
| No | 87.0% | 68.8% | NR (NA-NA) |  | 76.2% | 56.4% | 38.8 (28.2-49.4) |  |
| Liver meta. |  |  |  | <0.001 |  |  |  | 0.055 |
| Yes | 73.9% | 48.0% | 31.9 (19.9-43.9) |  | 54.4% | 36.3% | 24.3 (16.8-31.8) |  |
| No | 88.6% | 73.7% | NR (NA-NA) |  | 78.0% | 57.2% | 41.1 (31.3-50.9) |  |
| Bone meta. |  |  |  | <0.001 |  |  |  | 0.001 |
| Yes | 77.1% | 50.1% | 45.2 (27.0-63.4) |  | 67.9% | 30.6% | 27.3 (25.1-29.5) |  |
| No | 94.3% | 85.9% | NR (NA-NA) |  | 79.9% | 67.4% | 50.0 (38.0-62.0) |  |
| Brain meta. |  |  |  | 0.009 |  |  |  | 0.586 |
| Yes | 80.9% | 56.6% | 48.5 (26.8-70.2) |  | 75.2% | 49.3% | 33.6 (19.8-47.4) |  |
| No | 89.8% | 77.3% | NR (NA-NA) |  | 76.3% | 59.5% | 41.1 (30.5-51.7) |  |
| Type of brain meta. |  |  |  | 0.612 |  |  |  | 0.452 |
| Single parenchymal | 63.5% | 63.5% | 49.1 (NA-NA) |  | 72.2% | 49.5% | 26.5 (11.9-41.1) |  |
| Multiple +/- seeding | 83.2% | 54.2% | 36.6 (23.4-50.8) |  | 76.0% | 50.2% | 50.0 (20.9-79.1) |  |
| New lesion or aggravation of brain meta. | | |  | <0.001 |  |  |  | 0.383 |
| Yes | 70.0% | 47.1% | 29.4 (9.6-49.2) |  | 72.6% | 50.5% | 34.0 (24.6-43.4) |  |
| No | 89.8% | 75.1% | NR (NA-NA) |  | 78.3% | 58.2% | 52.9 (32.7-73.1) |  |
| Dose adj. for afatinib |  |  |  | 0.266 |  |  |  | 0.080 |
| Yes | 84.1% | 64.7% | NR (NA-NA) |  | 78.3% | 64.9% | 41.7 (30.4-53.0) |  |
| No | 90.4% | 83.1% | NR (NA-NA) |  | 74.1% | 40.5% | 38.8 (28.2-49.4) |  |

Data are presented as percentages for survival rates and months (95% confidence intervals) for overall survival, unless otherwise stated.

Patients in group A received sequential treatment with afatinib and osimertinib, while patients in group B received other therapies following first-line afatinib treatment.

**§** Tumor stage was classified based on 8^th^ edition of the American Joint Committee on Cancer staging system.

¶ Other tissue types included squamous cell carcinoma in 2 patients, adenosquamous cell carcinoma in 2 patients, and non-small cell lung cancer in 3 patients.

† Patients not presenting with EGFR Del19 and L858R mutations, including de novo T790M mutation, are classified as the “Others” group.

ECOG PS, Eastern Cooperative Oncology Group performance status; EGFR, epidermal growth factor receptor; Del19, deletion 19; meta., metastasis; adj., adjustment; CI, confidence interval; OS, overall survival; NR, not reached; NA, not-available.
